# Women’s Reasons for Leaving the Engineering Field

**DOI:** 10.3389/fpsyg.2017.00875

**Published:** 2017-06-30

**Authors:** Nadya A. Fouad, Wen-Hsin Chang, Min Wan, Romila Singh

**Affiliations:** ^1^Department of Educational Psychology, University of Wisconsin–Milwaukee, MilwaukeeWI, United States; ^2^Department of Management, McCoy College of Business, Texas State University, San MarcosTX, United States; ^3^Lubar School of Business, University of Wisconsin–Milwaukee, MilwaukeeWI, United States

**Keywords:** women engineers, attrition decisions, reasons for attrition, person-environment fit, occupational turnover, women’s career development

## Abstract

Among the different Science, Technology, Engineering, and Math fields, engineering continues to have one of the highest rates of attrition (Hewlett et al., 2008). The turnover rate for women engineers from engineering fields is even higher than for men (Frehill, 2010). Despite increased efforts from researchers, there are still large gaps in our understanding of the reasons that women leave engineering. This study aims to address this gap by examining the reasons *why* women leave engineering. Specifically, we analyze the reasons for departure given by national sample of 1,464 women engineers who left the profession after having worked in the engineering field. We applied a person-environment fit theoretical lens, in particular, the Theory of Work Adjustment (TWA) (Dawis and Lofquist, 1984) to understand and categorize the reasons for leaving the engineering field. According to the TWA, occupations have different “reinforcer patterns,” reflected in six occupational values, and a mismatch between the reinforcers provided by the work environment and individuals’ needs may trigger departure from the environment. Given the paucity of literature in this area, we posed research questions to explore the reinforcer pattern of values implicated in women’s decisions to leave the engineering field. We used qualitative analyses to understand, categorize, and code the 1,863 statements that offered a glimpse into the myriad reasons that women offered in describing their decisions to leave the engineering profession. Our results revealed the top three sets of reasons underlying women’s decision to leave the jobs and engineering field were related to: first, poor and/or inequitable compensation, poor working conditions, inflexible and demanding work environment that made work-family balance difficult; second, unmet achievement needs that reflected a dissatisfaction with effective utilization of their math and science skills, and third, unmet needs with regard to lack of recognition at work and adequate opportunities for advancement. Implications of these results for future research as well as the design of effective intervention programs aimed at women engineers’ retention and engagement in engineering are discussed.

## Introduction

Researchers, educators, policy makers, economists, psychologists, and leaders in technology fields have decried the underrepresentation of women in Science, Technology, Engineering, and Math (STEM) fields. Billions of dollars of federal funding over the past 20 years have focused on developing interventions to increase the pipeline of middle and high school girls entering STEM fields (White House Office of Science and Technology Policy, 2013). For example, in fiscal year 2011, nearly 3 billion federal dollars were spent on STEM education, roughly a third to support and encourage underrepresented groups to enter STEM fields. The result is that 18% of engineers graduating in 2011 were women, an increase from the 15% of graduates in engineering in 1990 (National Science Foundation [NSF], 2014). Despite the gains in female engineering graduates, only 11.7% of women are practicing engineers, a figure that has been relatively constant for two decades. Clearly, many women engineers are leaving the field of engineering. Even though they are attracted to the field, and complete a rigorous program of studies to be prepared as engineers, something occurs to either prevent them from entering an engineering career or leave the field after they enter.

There have been many attempts to understand why women leave the engineering profession (e.g., Buse et al., 2013; Singh et al., 2013;American Association of University Women [AAUW], 2015; Fouad et al., 2016). Some have argued that women are not confident enough to be engineers (e.g., Cech et al., 2011), others reported that women are vulnerable to stereotype threat (Murphy et al., 2007; Block et al., 2011), and engage in more social coping (Morganson et al., 2010), or that women are dissatisfied with their pay and promotional opportunities (Hunt et al., 2012). Other research pointed out that women engineers may contemplate departure when they perceive their work and occupational environment as not meeting their needs (American Association of University Women [AAUW], 2015). Blickenstaff (2005) reviewed the many explanations for women’s departure from a science career and concluded that many of these explanations have focused inappropriately on the need to remediate some aspect of girls and women. She argued that a series of solutions are needed, starting with a greater diversity in the workforce and emphasized the need to create an equitable workplace that would foster innovations that reflect diverse perspectives in design. Blickenstaff (2005) highlighted the need for changes to science education in order to increase the retention of women scientists, but her arguments are equally relevant for the field of engineering.

These studies provide valuable insights into the reasons why women may consider leaving their engineering workplaces and the engineering profession. However, with the exception of one study (Fouad et al., 2016) that compared women currently working in engineering with those that left the field, there is a paucity of research that understands the reasons why women engineers left the engineering profession. Just like “exit interviews” help human resource managers understand and address areas that need to be fixed within the organization, understanding the experiences of women engineers who *left* the profession can help shed light on workplace factors that were instrumental in their decisions to leave the field so that organizations can adequately address appropriate mechanisms to retain and engage women engineers.

We seek to make four contributions with this research. First, we use a national sample of women engineers from a variety of engineering disciplines that allows us to draw a comprehensive portrait of women engineers’ workplace experiences that foreshadowed their occupational departure decisions. Second, by uncovering the reasons for departure from the perspective of women engineers who already left the engineering field complements the insights generated from research on women engineers that are contemplating leaving the field. Third, we identify a comprehensive set of workplace factors that are theoretically implicated in individuals’ decisions to persist in, or depart from an occupation but have not yet been addressed in extant research. We do so by using a theoretical lens that allows us to capture the interface between individual and workplace factors that underlie persistence and departure decisions. Finally, we employ qualitative analysis that offers a rich supplement to the empirical research. By using qualitative analysis of comments from women engineers who recently departed from the engineering field, we are able to identify the factors that tipped their decision toward departure rather than persistence.

### Theoretical Background and Research Questions

Occupational turnover is costly, especially in fields like engineering that are characterized by rigorous education and training requirements. In addition, occupational turnover costs get amplified in fields like engineering that have a high cost of entry and exit, and when reentry is difficult (Ng and Feldman, 2007). Occupations, defined as “collections of work roles with similar goals that require the performance of distinctive activities as well as the application of specialized skills or knowledge to accomplish these goals” (Dierdorff et al., 2009, p. 974), have been shown to exert a “top–down” influence (Cappelli and Sherer, 1991) on a variety of individual level outcomes and experiences. In a series of studies, Dierdorff and his colleagues explain how occupations exert this “top–down” influence on individual behaviors and outcomes. According to Dierdorff and Morgeson (2013), any occupation encompasses several organizations, and jobs are embedded within the organization and occupation. They further note that occupations are characterized by their own “cultural features” (Morgeson et al., 2010, p. 353) and specific “occupational reinforcer patterns” (Dierdorff and Morgeson, 2013, p. 689) that promote or inhibit the satisfaction of needs and values of the employees working in the organizations. They also add that occupations enable or constrain the emergence of different work design elements that shape the manner in which work roles are enacted and jobs performed in any organization (Morgeson et al., 2010). Finally, they argue that occupational theories like the Theory of Work Adjustment (TWA) add unique value to our understanding of the links between occupational contexts and individual level outcomes because they “describe the influences occupational context exerts on individuals’ behavior and attitudes, and suggest that congruence between individuals and their environments can explain various outcomes such as job satisfaction” (Morgeson et al., 2010, p. 353) and in our case, occupational turnover decisions.

The TWA (Dawis and Lofquist, 1984; Dawis, 2005), may be useful in explaining why women leave engineering since it was specifically developed to understand factors involved in people’s decisions to leave an occupation or job (Juntunen and Even, 2012). The TWA belongs in the realm of person-environment theories within large class of occupational theories In general, person-environment fit theories capture the interface between individual and specific environment (job, organization, or occupation) factors that underlie the individuals’ satisfaction, adjustment, and/or fit with the environment and are manifested in their decisions to persist in or leave that environment. One person-environment fit theory in particular, TWA originally focused on the process of adjustment to work (Dawis and Lofquist, 1984; Dawis, 2005; Swanson and Schneider, 2013), but it is also useful in predicting whether individuals would be satisfied with their jobs (or occupations) and how long they might remain in the job (or the occupation). Person-environment fit within TWA is considered to be reciprocal. The occupation (or environment) has requirements of the individual (his or her ability to do the job), and the individual has requirements of the environment (the environment’s ability to satisfy his or her needs). According to TWA, if an individual’s needs are met by the reward (reinforcers) in the occupation, he or she is predicted to be satisfied and will stay in the occupation. Conversely, if there is little correspondence between an individual’s needs and the occupational reinforcers, and the individual cannot act to bring the two more in alignment, the individual will leave the occupation.

In terms of the person-environment fit framework, the TWA describes the person or the individual perspective through the construct of values and needs. Values are viewed as representing a grouping of needs. Dawis (2005) defined six crucial values: achievement, comfort, status, altruism, safety, and autonomy. Cluster analyses were used to identify three to six needs that corresponded to a particular value. *Achievement* values include using one’s abilities in the work environment and the job providing the individuals with a meaningful sense of accomplishment. *Comfort* values capture various aspects of the work environment that provide security, compensation, good working conditions, engaging work, variety in work, and ability to be independent. *Status* values reflect the occupation’s provision of opportunities for advancement, recognition, authority, and social status. *Altruism* values include having good relations with co-workers, doing things for other people, and doing work that does not feel morally wrong. *Autonomy* values include being able to be creative, having responsibility, and being able to be autonomous. Finally, *Safety* values reflect fair company policies, good supervisors who back up their workers and provide good training.

From the perspective of the environment, research underlying TWA has identified occupational ability patterns and occupational reward patterns in describing a wide variety of occupations. Early research on TWA established reinforcer patterns for STEM careers, including engineering (Rounds et al., 1981). For example, the occupation of engineering reinforces Achievement, Status, and Comfort. No studies have explicitly examined the STEM occupational reinforcers with women or their satisfaction with their engineering jobs and the engineering occupation, although a few studies have examined women’s departure from STEM careers. It is possible that women leave the engineering field because of a need-reinforcer mismatch. They may, for example, have other needs for reinforcers not provided by the environment, such as altruism or autonomy as was illustrated in a study by Trower and Chait (2002). They surveyed women faculty in over 130 institutions and found that female faculty in STEM have lower job satisfaction than their male colleagues. The researchers found that difficulty of fitting into a department, opportunities to work with senior faculty, and institutional support were the most significant factors that impacted women faculty’s level of job satisfaction. However, since this study only focused on women who worked in academic environments, the results may not apply to those who work outside of academic settings.

In a longitudinal study, Glass et al. (2013) examined women’s departure from STEM fields over time. They found that women in STEM-related occupations were more likely to leave their occupational field than women in other professions. The researchers found that demographic and family characteristics of women in STEM jobs were not saliently different from women in non-STEM professional jobs (Glass et al., 2013). Moreover, some protective factors such as higher earning, egalitarian gender attitudes, and better work-life support might prevent women from leaving their occupations temporarily but did not prevent women from moving out of the STEM fields over time. The authors argued that one possible reason that women in STEM occupations often move to non-STEM jobs and occupations is the chilly climate that they experienced in their working conditions and environment. This view is further corroborated in an extensive study by Hewlett and Luce (2005). Hewlett and Luce’s (2005) surveyed 2,493 Science, Engineering, and Technology (SET) professionals and conducted additional surveys of 1,910 men and women within three multinational companies and found that women SET professionals confronted a variety of “push” and “pull” factors that shaped their decisions to persist in or leave the field. The women SET professionals in their study reported that they loved their work, found it intellectually challenging and stimulating, and loved being able to use their hard-earned skills to make a contribution to society, these were the “pull” factors that kept these women highly committed and involved in their fields. Yet, these women faced extreme job pressures, narrow, murky career advancement opportunities, and often worked in workplace cultures, that were “at best unsupportive and at worst, downright hostile to women” (p. 7) that thwarted their attempts to succeed and thrive and “push” them out of the workplaces. Viewed in terms of the TWA, it is possible that there is a misalignment or a mismatch between the women’s needs from their work environments and occupations and what their environment (job and/or occupation) offered and provided them.

Although these previous studies provided valuable information about the differences between women in STEM fields and non-STEM fields and indicated that promotion, higher job satisfaction, and job commitment might help companies keep their female workers, little is known specifically about whether women engineers’ actual working conditions and experiences may underlie a “need-reinforcer” mismatch and act as a potential trigger for them to quit the engineering field. Using the TWA, we apply the lens of the six values and their corresponding needs to delve into, and categorize, the match/mismatch between the working conditions experienced by women engineers who worked in engineering and the needs they sought to satisfy in their workplaces. Such an approach will allow us to not only develop a deeper understanding of the key areas of person-environment match/mismatch but also help us document the different types of reasons and circumstances that precipitated a large group of women engineers to leave the engineering field after pursuing an engineering career. Given that there is little empirical literature and limited conceptual and theoretical guidance in this area, we offer the following research questions:

*Research Question 1*: Are women engineers’ decisions to leave their jobs and the engineering field driven by a mismatch between their occupational needs and the reinforcers provided by the work environment?

*Research Question 2*: Will women engineers’ attrition decisions be characterized by the same reinforcer pattern of values and needs of Achievement, Status, and Comfort as described by Rounds et al. (1981)?

*Research Question 3*: Will women engineers’ attrition decisions be characterized by other reinforcer pattern of values and needs not captured by Rounds et al. (1981)?

## Materials and Methods

### Procedure

The data were collected through an online survey in 2009. A survey link was sent to 70 universities with engineering departments across the United States. These universities were chosen from an annual list compiled by the American Society for Engineering Education (ASEE) that profiles the universities based on their record of graduating the most women engineers for that year; our list was based on ASEE compilation of 2008. Out of the 70 universities that were contacted, 30 universities and their engineering departments formally partnered with the researchers. The women engineering alumnae from these 30 universities were sent a link to the website; only those who agreed to the informed consent were given access to the survey.

Links to the survey website were also sent by these women alumnae to their female engineer colleagues, friends, and family members. A total of 5,562 women who had a bachelor’s degree in engineering participated in this survey after completing the informed consent form.

### Sample

About 10% (554) of these women never entered the field of engineering, 27% (1,464) chose to leave the engineering field, and 60% (3,324) were still working in engineering in 2009. In this qualitative study we focused on the comments of the 27% of the participants (*N* = 1,464) who left the profession of engineering having worked in engineering workplaces for a period of time.

Of the 1,464 women participants who chose to leave the engineering field, 65% identified themselves as white, 4% as Asian/Asian American, 3.8% as African American, 2% as Latina, 22% as Multi-racial, 1% as “other,” and less than 1% self-identified as Native American. Regarding their marital status, 66% of women in this group were married, 23% were not married, 4% were in a committed relationship, 1% reported having separated from their spouse/partner, 1% were divorced, and 1% reported being widowed. About 60% of women in this group were parents. In addition, 19% of the women (279) left the engineering field less than 5 years ago. For this group, the average age of women ranged from 19 to 66 with a mean age of 35 years.

For other women who left engineering after having worked in this field, their ages ranged from 21 to 85 years. Almost half of these women reported having a full-time employed spouse. About 42% of women who left the engineering field reported at least 40 working hours per week in current non-engineering positions.

The annual salary reported by women who left the engineering field ranged from less than $ 25,000 to more than $ 250,000. Twenty-three percent of women reported an annual salary of less than $ 25,000, and 26% of participants reported an annual salary between $ 51,000 and $ 100,000. In the sample, 26% of women who left the engineering field reported that their total family income was higher than $ 151,000 per year.

The top five majors reported by the women who left the engineering field were: Industrial Engineering (20%), Mechanical Engineering (18%), Electrical Engineering (13%), Chemical engineering (13%), and Civil Engineering (11%). Among this group of women, 42% pursued an additional educational degree: 27% earned a M.S., 12% earned a MBA, 9% earned a B.S., 3% earned a M.A., and nearly 2% earned a Ph.D. Additionally, the top four reasons why these women chose to leave the engineering field were work-family imbalance (16%), loss of interest in engineering work (12%), lack of opportunities for advancement (11%), and dislike of engineering tasks (9%).

### Survey Questions

The survey questions included both Likert type questions as well as open-ended questions. We used the responses to the two open ended questions for qualitatively analyzing that data and reporting the results in this paper. The two questions that were used for analyses were: what was their opinion and experience on why women leave engineering and what were their own experiences in engineering that they would like to share with researchers. Guided by TWA theory, the responses to the two questions were coded into six categories and used in the present study. Seven researchers from both a management department (one faculty member and one doctoral student) and a counseling psychology department (one faculty member and four doctoral students) coded the comments. In particular, each doctoral student coded 100 pages on average (with 3–6 statements on each page) and each faculty member coded 30 pages, and 10 pages overlapped. Thus, approximately 200 statements were coded by multiple raters. Any discrepancy in coding for a statement was flagged for discussion. After comparing the coding between researchers and doctoral students, we flagged 3% out of 1,863 comments and the discrepancies were reconciled.

## Results

The participants’ comments were grouped into categories of mismatch between their values related to *Achievement* (needs related to using abilities and achievement); *Comfort* (needs related to activity, independence, variety, compensation, security, and working conditions); *Status* (needs related to advancement, recognition, authority, and social status); *Autonomy* (needs related to creativity, responsibility, and autonomy); *Altruism* (needs related to co-workers, social service, and moral values); or *Safety* (company policies, supervision-human relations, and supervision-technical). The factors, below, and in **Table 1**, are discussed in order of most frequently identified values (Comfort) to least (Autonomy).

**Table 1:** Reinforcers, description, and number coded for women who left engineering.

Reinforcer	Description	Number of comments coded
Comfort^∗^	Needs related to activity, independence, variety, compensation, security and working conditions	699
Safety	Needs related to company policies, supervision-human relations, and supervision-technical	383
Achievement^∗^	Needs related to using abilities and achievement	282
Status^∗^	Needs related to advancement, recognition, authority, and social status	252
Altruism	Needs related to co-workers, social service and moral values	239
Autonomy	Needs related to co-workers, social service and moral values	38

*^∗^Reinforcers found in engineering occupations (Rounds et al., 1981).*

### Comfort

It comments were the most frequently identified as contributing to the needs-reinforcers mismatch in terms of how employees perceived their work environment, specifically the needs related to working conditions, pay, and security. There were 669 comments coded to the “Comfort” category of values.

Within the Comfort category of values, and the “working conditions” set of needs, those relating to work life imbalance stood out in their frequency. Many women reported that the engineering job was a busy one, to the point that it impacted their work-life balance. Many women reported that engineering jobs could be very demanding and “most companies expect +40 h.” They reported being challenged to be able to work fulltime when they had small children at home. For example, one participant stated that “I often had meetings scheduled outside of childcare hours, had travel expectations, and heavy workloads were not decreased. Eventually I went to working part time as a consultant, working to support tests and launches primarily for satellites.” The majority of women who left the engineering field stated that it was difficult for them to find part-time jobs in the engineering field, and that was the main reason they left the occupation altogether. However, it needs to be noted that not all of the women who left the engineering field had the same experience. A few had different experiences that related to their dissatisfaction with the nature of the work itself. One of these women commented, “sitting in front of a computer screen is fine and dandy when I have meaningful work, but I didn’t have meaningful work that kept me busy 40 h a week.” This particular respondent felt so strongly about the lack of meaningful work that she mentioned this was the only reason that made her decide to leave engineering.

Compensation was also an issue that was brought up by women engineers who left engineering. Some of them stated that the compensation they earned was not enough for them to pay childcare. As a result, it made sense for some them to quit their jobs and stay home to take care of their children on their own. For example, one of the participants shared that the “cost of childcare was very high... In my case, it became financially illogical to continue working, and my job satisfaction alone was not enough to keep me there.” Still others noted that they realized that people usually had better compensation in a business position than in an engineering position and that pay discrepancy drives many to leave engineering. Many women engineers specifically mentioned a concern with the possibility of glass ceiling and stated that they were not paid equally compared to their male counterparts and noticed “a gap in pay between men and women at the same skill level.” One respondent commented that having a lower salary than her male colleagues made her think about moving to another field to make more money. Another woman stated, “the career path in engineering does not allow for comparable income or management growth. There was a choice [for me] between further specialization and returning to an MBA.”

Some women expressed that the engineering field could not provide them with steady employment. For example, one of the participants shared that “I never felt like I had job security because of layoffs and the threat of layoffs.” Similarly, another stated, “I personally do not feel there is enough security working in industry.” As noted above, there were only limited opportunities for part-time work in an engineering field for those who need to have more flexible working schedule. Furthermore, many women stated that it was “hard to take a break and then come back” to the engineering field. One of them stated, “leaving engineering and trying to come back is not an easy feat. That is why many women who take a break to care for their children, never return.”

Working conditions also influenced individuals’ decisions to not work in engineering anymore. For example, one of the participants shared, “I worked for an organization that was an old boys club. The industrial nature of the job was dirty, smelly, and hazardous. This company had been run by white men for so many years that these guys had no idea how to integrate women into their organization.” Similarly, another woman stated, “the physical work environment in manufacturing is often loud, hot, cold, dusty, smelly, etc., and in early assignments, shift work or being on call may be required.”

### Safety

The second set of unmet needs was related to whether the organization’s policies and practices were fair. There were 383 comments coded in this category. Even though some women reported that they had good relationships with their co-workers and liked the engineering tasks they were doing, the lack of mentoring support combined with discrimination from their supervisors, formed the key reasons in their decision to leave the field.

Many women talked about the need for female mentors and role models in the field. For instance, one of them wrote, “The biggest problem I experienced was lack of a female mentor. My last 2 years working as an engineer, I finally found a female mentor, however, she was the comptroller of the company, not an engineer. I had no female to whom I could look up to.” Echoing a similar sentiment, one woman noted “It was also hard without having female mentors in the field. It would have helped to have someone to talk with about issues.” One of the participants stated that she loved being an engineer, and “if I had had a female mentor, I may have stayed. But I felt very alone and didn’t feel encouraged or inspired to continue.” Assisting women engineers finding/ matching with female mentors could be critical to help them stay in the field.

Flagrant violations of some HR policies also contributed to women’s decisions to leave the field of engineering. One of them disclosed that her boss suggested that she should sleep with their customers, and when she refused to do so, her boss filed sexual harassment claims on her. Some women reported that their bosses did not support them when they needed to have maternity leave or requested more flexible work schedules. For instance, one participant shared that her boss told her she would never return once she went on maternity leave. Others stated that their bosses were unwilling to let them work part-time. Still another woman tried to discuss with her boss when her job had increased to 70% of the time spent on traveling, including overnight travel. She stated that “I tried to transfer to a different position within the company and was unable to. My boss was totally unwilling to try and work out a compromise on the travel so I left.”

Violation of other company policies also contributed to the woman engineers’ decisions to leave the field. For example, one of the participants reported, “I found that ethical concerns were not well-addressed, and the company was more interested in papering over problems, often at very high expense, than actually solving them.” Failure to accommodate disabled individuals was also noted as a factor that pushed some women away from the engineering field. For instance, one woman shared, “I am disabled and needed disability accommodations. I requested them and was turned down. In addition, my management purposely took away the few accommodations that I had received under another manager (like flexible working hours). I was put on display and required to regularly go into inaccessible rooms in order to do my job.”

### Achievement

The third most frequent set of unmet needs focused on needing to feel confident in using one’s abilities and having meaningful work. How this mismatch between needs and environmental reinforcers influenced women’s decisions to leave the engineering field can be found in 282 comments. Many women engineers left the field because they wanted to work in other environments that better utilized their abilities. For example, one of the participants stated that even though she was a top ranked engineer when she was working in the field and enjoyed engineering work, her passion in her life was teaching math and science to students. As a result, she decided to leave the engineering field. Similarly, another woman chose to switch to another field because the new field allowed her to use “the analytical, research, and writing skills that I developed during my engineering training and work, as well as use my compassion, creativity, multidisciplinary thinking, and cross-cultural values and skills.” There were also some women who decided to change their career path because sometimes working in the engineering field was not the ideal place for them to apply their math and science skills. For instance, one of the women who loved math and science found that the engineering jobs she had “involved no math and little science.” This became a factor that pushed her out of the field.

Some woman left the field because the engineering job did not give them a feeling of accomplishment. For example, one of the participants shared that a “big killer” in her engineering job was that she “learned nothing.” Echoing her point of view, another participant reported that engineering “just wasn’t a very fulfilling profession” and she also decided to leave the field. Another woman stated that she found the engineering culture unsatisfying and felt much more fulfilled after she changed her career to work in another field.

### Status

Lack of recognition was another factor that pushed women out of the engineering field. There were 252 comments that fell into this category. It appears that some women did not feel they were being respected or received enough recognition in their work. For instance, one woman commented, “I could expect very little recognition, both financially and organizationally.” Similarly, another participant stated that people often told her that she “had to work twice as hard to get half the recognition.” In addition, one of the participants stated, “Excellence in engineering is not rewarded with promotions, raises, or even appropriate recognition.”

Another significant factor that many women highlighted was related to the lack of opportunity for advancement. Some of them encountered challenges in terms of getting promotions in a male dominated field and felt that “that the climb up the ladder of advancement is littered with constant obstacles.” Many of them expressed that other fields offered more advancement opportunities than the engineering field. For instance, one of them said that there are “more perceived opportunities for advancement in the business world.” Some of them reported that they decided to leave engineering “not because of a dislike for engineering, gender discrimination, or other such inequality,” but because “the opportunities for advancement (and financial gain) were greater [elsewhere].” Some women reported that they were promoted out into other fields. For example, one of them reported that “I left operations to become manager of the commercial group and whereas my engineering background was useful, it wasn’t the main skill necessary to perform the job. I really liked Operations but in order to get promoted, one had to move up and out of Operations.”

### Altruism

The fifth set of unmatched needs was related to employees’ relationships with their co-workers, doing things for other people, and doing work that is consistent with their moral values. There were 239 comments that were related to this category. Some women reported that they “wanted to work with people with social skills.” One of them stated, “I left my job not because of dissatisfaction with engineering, but the people I worked with.” Many of the participants talked about a “boys club” mentality that still exists in engineering. For example, one of the women shared, “There is still an “old boys club” where women are excluded from “male-bonding” events (poker games, golf, etc.). This informal channel of information is often where we can learn more about our managers and job expectations but puts us at a disadvantage because we aren’t even invited to participate.” Some decided to leave the engineering field because they wanted to feel like they were doing things for other people. For example, one of the women stated that she “wanted to feel the work I was doing mattered to my community.” Another woman chose to change her career path and focus on improving healthcare, stating that’s an area that “truly contributes to society.”

### Autonomy

The final set of needs refers to employees’ needs to be autonomous. This category had the least number of comments; only 38 comments fell into in this domain. One of the women who left the engineering field shared that she wanted a change and wanted to have “more leadership/management roles, roles where there are more women, roles with more opportunities for growth, getting more broad experiences rather than depth in one area.” Some chose to switch their career path because they wanted to try out the viability of some of their own ideas. For instance, one woman who left engineering stated, “I was bored (of designing the same product over and over, of reacting to the same mfg [manufacturing] defects, of working with the same rough clients, and when I finished my MBA, I went into management consulting.”

Our first research question posed whether women engineers’ decisions to leave their jobs and the field of engineering will be shaped by the need-reinforcer mismatch as posited by the TWA. Our results reveal that women’s decisions to leave engineering reflected that the work environment did not fulfill their occupational values. Our second research question posed whether the occupational reinforcer values of Achievement, Comfort, and Status would characterize women engineers’ decisions to leave the field. Although, we found the same three dominant values emerge in women’s rationale for leaving engineering, the pattern we found (of Comfort, Achievement, and Status) was a little different from what Rounds et al. (1981) initially described. Finally, the last research question asked whether there will be other unmet needs that characterize women’s decisions to leave the engineering field, and we found that unmet needs related to safety and altruism did play a likely role in women’s decisions to leave their engineering work and field.

## Discussion

The purpose of this study was to provide a deeper and more comprehensive understanding of the reasons women engineers leave the field than what has been previously known. Using the TWA (Dawis, 1994), we undertook a qualitative analysis of comments made by 1,464 women engineers reflecting a wide variety of work experiences and conditions that factored into their decisions to leave their jobs and the engineering field. According to the TWA (Dawis, 1994), departure from a work environment is due to a mismatch between the needs an employee has and the reinforcers provided by the work environment in the organization, which is embedded within the occupation. TWA proposes six overarching needs: comfort, safety, achievement, status, altruism, and autonomy. The reinforcers found in engineering occupations are related to achievement, status, and comfort. We suggested that women would be more likely to leave an engineering occupation if their needs in these areas were not reinforced by the work environment. Overall, our results provide definitive answers to the three research questions.

The first research question posed whether women engineers’ decisions to leave their jobs and the engineering field is driven by a mismatch between their occupational needs and the reinforcers provided by the work environment. As the results and comments reveal, there are clear indications that women’s decisions to leave their jobs and the engineering field was shaped by a lack of fit between their needs and values and the reinforcements present in their work environment. The second research question asked whether women engineers’ attrition decisions will exhibit the same reinforcer pattern of values and needs of Achievement, Status, and Comfort as described in Rounds et al.’s (1981) original study. Our results show that occupational values and needs related to Comfort, Safety, and Achievement predominantly characterized women engineers’ attrition decisions, and this pattern closely paralleled the one suggested by Rounds et al. (1981). The final research question centered around the possible presence of a different reinforcer pattern of values than the one original characterized by Rounds et al. (1981). Our results showed the emergence of another set of occupational reinforcers related to status, altruism, and autonomy needs. These three needs were not identified by Rounds et al. (1981) in their original work on engineers. The following paragraphs discuss the meaning and implications of these findings.

Comfort needs emerged as the most dominant category of occupational needs that were misaligned with the reinforcers in the work environment and this mismatch undergirded women engineers’ decisions to leave their workplaces and the field. A third of the comments were related to unmet comfort needs that were associated with perception of poor and/or inequitable compensation, poor working conditions, as well as an inflexible and demanding work environment that made it difficult to balance work and family roles. Many participants noted that it was challenging for women with young children at home to persist in the engineering field because of the heavy workload and travel expectations. However, many women also noted that they tried to find ways to actively alter the work environment to bring those needs more in alignment, but were not successful in advocating for part time work or flexibility in their jobs.

Unmet safety needs took the form of unfair, and sometimes illegal, organizational practices and policies. From the comments it appears that many women engineers experienced discrimination from their supervisors or sexual harassment but the system of filing a discrimination charge was not functioning well in the company they worked with at that time. In addition, the comments point to unmet safety needs in the form of non-existent systems and practices that would increase employees’ engagement in the workplace. Specifically, we found that women engineers’ unmet needs for mentoring in the work environment increased their feelings of isolation and made it hard to aspire for further advancement in the company.

These deeply troubling and unsatisfying job experiences are analogous to the “push” factors that Hewlett and Luce (2005) describe in their study on female SET professionals’ decision to take the “career off-ramps” when confronted by hostile macho cultures, extreme job pressures, isolation in the work environment, and murky career advancement paths. These “push” factors were strong enough to override the “pull” factors that these women reported in terms of loving their work for its stimulating, intellectually challenging aspects and for being able to use their skills to make a difference in the world. Similar to the Hewlett and Luce (2005) female SET respondents, the women engineers in our study also left the field of engineering because they wanted to utilize their skills in another field; these comments reflected their unmet achievement needs. Some chose to study engineering because of suggestions from family or school counselors or because they were good at math and science, and eventually the unmet need to more effectively use their math and science skills than the current environment offered, led them to another career. Some participants also shared their eagerness to learn new things and contribute to the community, both of which were not being addressed by their current work environment.

Finally, many women described unmet needs linked to status, which reflected their needs for recognition and opportunities for advancement. Comments related to status needs related to their dissatisfaction with fewer advancement opportunities than their male co-workers and lack of recognition by their supervisors. This finding was consistent with the trends reported by Powell et al. (2009) in their study on women engineers.

Thus, as predicted, many women left engineering because the environment did not provide them the opportunity to meet their needs primarily related to comfort, safety, and achievement, despite their attempts to bring the needs and reinforcers more in line with each other. According to the TWA, the women’s descriptions of attempts to change policies, talk with supervisors, or make modifications to their work environment, are considered attempts at active adjustment, that is, acting on the environment to reduce dissatisfaction. As the theory would predict, when those attempts fail, the individual will choose to leave the environment altogether, which is reflected in the pattern of findings from our study.

Cumulatively, our results indicate that fulfilling one’s needs related to challenge, autonomy, and a sense of balance, parallels the same three dominant needs within the kaleidoscopic model (Maineiro and Sullivan, 2005) that emerge at different points in women’s lives and careers. However, unlike Maineiro and Sullivan’s (2005) model, and research by O’Neil and Bilimoria (2005), the current study did not take a longitudinal or a ‘phased’ view of women’s careers.

The women in our group also noted two additional types of reinforcers that were not met in the engineering organizations they work for: safety and altruism. Safety comments were primarily related to lack of supervisor support and the unfair application of policies. Many women noted that the “boys club” culture in the engineering field still exists and many participants in our study stated that it made them feel like an outsider. These comments are consistent with other reported research on women’s experiences in STEM occupations (Farrell, 2002; Gupta and Sharma, 2003; Hewlett and Luce, 2005). The lack of altruism needs indicated that working in engineering did not require a lot of interpersonal interactions. Some women engineers decided to change their career to allow them more opportunities for interpersonal interactions.

### Practical Implications and Suggestions for Future Research

According to O’Neil et al. (2013, p. 103), “Career development occurs at the intersection of the individual and the organization.” We contend that to the extent that organizations are the vehicles through which the occupational reinforcer patterns and values are made manifest, then career development may be viewed as occurring at the intersection of the individual, organization, and the occupation. In highlighting the person-environment fit issues that undergird women’s decision to leave the engineering profession, it is important to place the results from this study within the larger context of women’s career development. Our results revealed that women’s individual competence and ability to connect with others is a significant factor in their career decision-making and progression and it parallels one of the patterns that O’Neil et al. (2008) identified in their overview of research on women’s careers. The results also indicate that beyond the needs for competence, autonomy, challenge, and balance, women’s career decisions are also shaped by issues of inequity – in compensation, advancement opportunities, and interpersonal treatment (in the form of harassment and discrimination). These issues were somewhat reflected in O’Neil and Bilimoria’s (2005) characterization of the “pragmatic endurance” career phase which described women’s perseverance through demanding organizational environments. Future models of women’s career development need to more explicitly take into account the role of workplace inequities in shaping women’s career choices and their overall career trajectory. In addition, we encourage researchers to employ inductive approaches to build theory that captures the complex interplay of occupational, organizational, and personal factors that shape technically skilled women’s career choices and career trajectories.

Based on our findings, there are some suggestions that might be useful for employers and educators. First of all, women left engineering even though their needs were in line with the needs provided by the occupation, but not adequately reinforced through the organizations’ work design, systems, and practices. In other words, engineering is a field that reinforces achievement, comfort, and status, and yet women expressed dissatisfaction with the lack of reinforcers in those areas as it manifested through the organizational practices and cultures. This would seem to be an area for intervention by employers, interventions that provide career development opportunities for employees, a sense of accomplishment at work, security, good compensation, good working conditions, and opportunities for advancement.

Many of the comments related to comfort focused on frustrations with the ability to manage multiple roles. Although many of participants’ organizations had work-life benefits, women noted they were actively discouraged from using them. Clear communication from organizational leaders supporting the use of work-life benefits would be helpful as also clear messaging dissuading supervisors from imposing career penalties on women for using any work-life benefits. Having flexible work schedules, creating more part-time opportunities, or allowing employees to work remotely could also be beneficial for engineers who have care-giving responsibilities.

Organizational and HR leaders also need to send a clear message regarding a work environment that is free from harassment and discrimination and policies that reflect fair, equal, and respectful treatment of all individuals at work. Many of our participants stated that it would be helpful for them to have a woman supervisor or mentor to discuss the challenges they face in their work environment. Given the paucity of women engineers at all organizational levels, it might be challenging to fulfill this particular need, but companies could train their male supervisors to become more sensitive and responsive to challenges that their women engineers face.

Based on our results, companies could also train supervisors on how to provide positive feedback and encourage their subordinates. It might be helpful for companies to provide more training opportunities for their employees so that they can learn new skills and knowledge to apply in their work. Companies could create some social events to increase cohesiveness among employees. It might also be beneficial to let employees know why their work is important and how it could benefit other people in the community. In addition, organizations could create volunteer opportunities for their employees to partner with community members on mutually meaningful activities and projects.

Even though our study provides more detailed explanation of why women engineers leave the field, there are some limitations that need to be acknowledged and which serve as potential areas for future research to investigate. First of all, we did not directly ask women engineers the different things they needed to adjust in their work environment. This could be valuable for future researchers to find out what do women engineers need to cope with the challenges they face. It would be also important to document the successful strategies used by women engineers who have persisted in the engineering field and draw lessons from their efforts to actively alter their work environments to meet their occupational needs. Toward that end, scholars need to build new theories, refine existing theories, and/or integrate complementary theoretical frameworks to offer a more nuanced understanding of this phenomenon. Our use of the TWA framework provides some compelling new insights, explanations, and interpretations of this area of inquiry but there are additional theoretical and methodological ways to approach this multifaceted phenomenon. Second, since we solicited written responses to open ended survey questions, we did not have an opportunity to clarify the meaning of their comments. Future researchers should be encouraged to use multiple methods to capture women’s work experiences and not be limited to one method of data collection. The final limitation that needs to be noted is the sample used in our study. Although we argued that it is important to capture the women engineers’ voices and experiences, we emphasize that it is equally critical to understand male engineers’ perspectives and work experiences that undergird their decisions to continue in, or leave, the field of engineering. In fact, it will be interesting to see whether some of the mismatch needs found in our study of women engineers also is an equally relevant driver of male engineers’ decision to leave the field. As noted by Emslie and Hunt (2009), male engineers have been facing similar dilemmas of work-life balance. We encourage future research to explore whether TWA theory will be helpful in explaining the departure and persistence decisions of male engineers.

## Conclusion

Our research was broadly anchored with the person-environment fit theoretical framework and we used the TWA (Dawis and Lofquist, 1984) to shed light on a little known facet of women engineers’ work experiences, i.e., the degree to which the work environment fulfilled and matched women engineers’ occupational needs, and the extent to which these unmet needs triggered their decisions to leave their engineering jobs and the occupation. Lee et al. (1999) noted that “Individuals experience unique circumstance when they leave” (p. 450) and our results contributed to the growing literature on understanding the drivers of occupational turnover decisions by uncovering some of these “unique circumstances” that galvanize women engineers to leave the engineering field that they worked and trained hard to enter.

## Ethics Statement

The study was carried out in accordance with the recommendations of the Institutional Review Board at the University of Wisconsin-Milwaukee. The research protocol was approved by the IRB at the University of Wisconsin-Milwaukee.

## Author Contributions

NF and RS designed the study and collected the data; NF took the lead in writing significant parts of the manuscript; RS shared part of the writing. W-HC and MW took the lead in data cleaning and analysis, writing the methods and results section. All authors were involved in coding and resolving discrepancies. All authors contributed substantially to the final manuscript.

## Conflict of Interest Statement

The authors declare that the research was conducted in the absence of any commercial or financial relationships that could be construed as a potential conflict of interest.
